# Synthesis, In Vitro, In Vivo and In Silico Antidiabetic Bioassays of 4-Nitro(thio)phenoxyisobutyric Acids Acting as Unexpected PPARγ Modulators: An In Combo Study

**DOI:** 10.3390/ph15010102

**Published:** 2022-01-15

**Authors:** Blanca Colin-Lozano, Héctor Torres-Gomez, Sergio Hidalgo-Figueroa, Fabiola Chávez-Silva, Samuel Estrada-Soto, Julio Cesar Almanza-Pérez, Gabriel Navarrete-Vazquez

**Affiliations:** 1 Facultad de Farmacia, Universidad Autónoma del Estado de Morelos, Cuernavaca, Morelos 62209, Mexico; clbi_ff@uaem.mx (B.C.-L.); Hector.Torres-Gomez@hki-jena.de (H.T.-G.); sergio.hidalgo@ipicyt.edu.mx (S.H.-F.); facasy@gmail.com (F.C.-S.); enoch@uaem.mx (S.E.-S.); 2 Facultad de Ciencias Químicas, Benemérita Universidad Autónoma de Puebla, Puebla 72000, Mexico; 3 Leibniz Institute for Natural Products and Infection Biology, Hans Knöll Institute, 07745 Jena, Germany; 4 CONACyT, Instituto Potosino de Investigación Científica y Tecnológica, San Luis Potosi 78216, Mexico; 5 Laboratorio de Farmacología, Departamento de Ciencias de la Salud, Universidad Autónoma Metropolitana Iztapalapa, Mexico City 09340, Mexico; jcap@xanum.uam.mx

**Keywords:** PPAR, drug design, diabetes, molecular dynamics

## Abstract

Four isobutyric acids (two nitro and two acetamido derivatives) were prepared in two steps and characterized using spectral analysis. The mRNA concentrations of PPARγ and GLUT-4 (two proteins documented as key diabetes targets) were increased by 3T3-L1 adipocytes treated with compounds **1**–**4**, but an absence of in vitro expression of PPARα was observed. Docking and molecular dynamics studies revealed the plausible interaction between the synthesized compounds and PPARγ. In vivo studies established that compounds **1**–**4** have antihyperglycemic modes of action associated with insulin sensitization. Nitrocompound **2** was the most promising of the series, being orally active, and one of multiple modes of action could be selective PPARγ modulation due to its extra anchoring with Gln-286. In conclusion, we demonstrated that nitrocompound **2** showed strong in vitro and in vivo effects and can be considered as an experimental antidiabetic candidate.

## 1. Introduction

**Figure 1:**
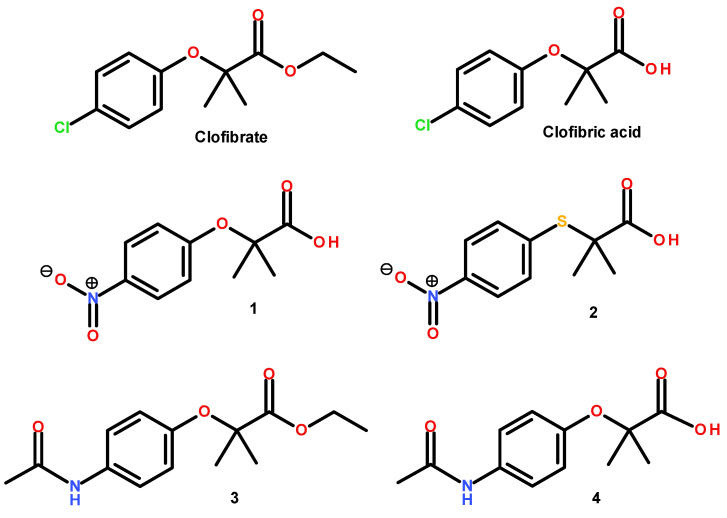
PPARα modulators clofibrate and clofibric acid, containing phenoxyisobutyric acid pharmacophore, and the nitrocompounds (**1**–**2**) and acetamide compounds (**3**–**4**) designed in this work.

Phenoxyisobutyric acid derivatives are a class of antihyperlipidemic agents, commonly known as fibrates, that act mainly as peroxisome proliferator-activated receptor (PPAR) modulators [1]. PPARα and PPARγ are the protein targets of several endogenous fatty acids, which function as antidyslipidemic and insulin-sensitizing molecules [2]. In addition, PPARγ controls target genes involved in numerous biochemical pathways, such as the glucose transporter GLUT-4 [3]. Clofibrate, the first therapeutically useful fibrate, is an ethyl chlorophenoxyisobutyrate that acts as a prodrug, which is metabolized in vivo by esterases into its active metabolite, clofibric acid, that has shown strong hypolipidemic effect [4]. Recent studies showed that clofibrate and clofibric acid exerted a potent inhibitory activity against the enzyme 11β-hydroxysteroid dehydrogenase type 1 [5]. This enzyme catalyzes the conversion of inactive cortisone into the active hormone cortisol, a powerful glucocorticoid that acts as a contender of insulin action and stimulates gluconeogenesis in liver, leading to an increase in blood glucose levels and causing hyperglycemia [6]. Type 2 diabetes, a metabolic complication represented by hyperglycemia, is originated by insufficiency in production or action of insulin [7]. In our current research on antidiabetic compounds, we explain here the preparation of isobutyric acid derivatives **1**–**4** (Figure 1)**,** their in vitro actions on PPARα/γ and GLUT-4 mRNA expression levels, the predictive biosimulations of their pharmacodynamics behavior and their in vivo effect in a murine model of diabetes. The collection of in vitro evaluations combined with in silico and in vivo estimations leads to the concept of in combo screening in antidiabetic drug discovery [5].

## 2. Results and Discussion

### 2.1. Chemistry

**Figure 2:**
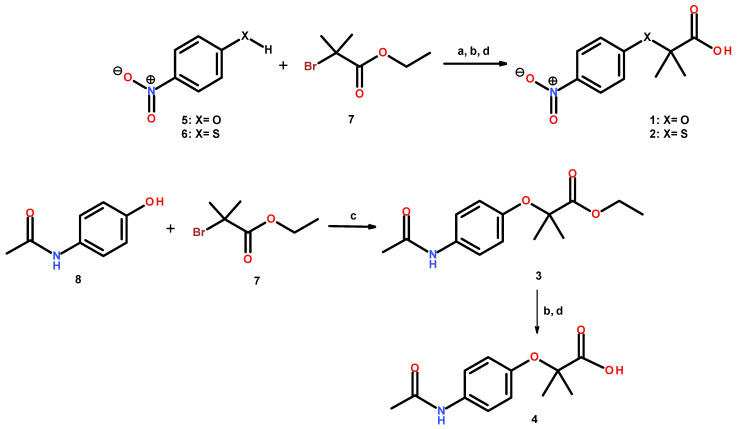
Synthesis of compounds **1**–**4**. (a) K_2_CO_3_, CH_3_CN, reflux; (b) LiOH, THF/MeOH/H_2_O; (c) K_2_CO_3_, DMSO, 80 °C; (d) HCl (10% *v*/*v*).

The preparation of compounds **1**–**2** is described in Figure 2. Nucleophilic substitution of ethyl 2-bromo-2-methypropionate (**7**) with 4-nitrophenol (**5**) or 4-nitrothiophenol (**6**) in basic conditions and with acetonitrile as a solvent afforded the correspondent ethyl ester precursors as oil products. The ethyl esters formed were treated immediately with a mixture of THF/MeOH/H_2_O (3:2:1) and hydrolyzed with five equivalents of LiOH to obtain compounds **1** and **2** in moderate yields (62.8 and 58.9%, respectively). Compound **3** was produced in a similar way, employing 4-acetylaminophenol as a nucleophile in dimethylsulfoxide in basic media. A white solid was obtained in moderate yields (44%). The subsequent selective ester hydrolysis of **3** with lithium hydroxide afforded **4** in 83.13% yield (Figure 2). All the reactions were monitored by thin layer chromatography (TLC), and the products were separated by filtration or extracted with CH_2_Cl_2_, affording the corresponding compounds **1**–**4**, which were recovered with 44–83% yields and purified by recrystallization with adequate solvent, as mentioned in the Section 3. Chemical structures were established by spectroscopic (^1^H, ^13^C NMR) and spectrometric analysis. Purity was ascertained by microanalysis.

In the ^1^H NMR spectra, the signals of the respective protons of the compounds were assigned, detecting the chemical shifts, multiplicities, and coupling constants (*J*). All molecules exhibited a single signal ranging from δ_H_ 1.46 to 1.71 ppm, attributed to a geminal dimethyl group. All compounds displayed characteristic signals of 1,4-disubstitued benzene. The aromatic region of the ^1^H NMR spectrum of compounds contained an A_2_B_2_ pattern signal ranging from δ_H_ 6.75 to 7.65 ppm (d, *J*_ortho_ = 8.7–9.6 Hz) and 7.45 to 8.17 ppm (d, *J*_ortho_ = 8.4–9.6 Hz), attributed to the equivalents H-2′, H-6′ and H-3′, H-5′ hydrogens, respectively. The displacement for carboxylic protons in **1**, **2** and **4** was found between 9.83 and 10.6 ppm as a singlet. For compounds **3** and **4,** a singlet signal for acetamide hydrogens was found ranging from δ_H_ 2.0 to 2.1 ppm. For the ^13^C nuclear magnetic resonance spectra, constant signals were found for the benzene nucleus in all compounds, one signal ranging from δ_C_ 24.5 to 25.7 ppm, attributed to a geminal dimethyl group. An additional signal ranging from δ_C_ 51.5 to 79.8 ppm was assigned to C-2 of the ether bridge. In the aromatic region of the spectra, signals from 118.2–120.3 ppm and 120.2–136.1 ppm were assigned to C-2′, C-6′ and C-3′, C-5′, respectively. Other frequent signals were found in downfield shifts from δ_C_ 133.7–145.4 ppm, assigned to C-4′, 140.6–161.8 ppm assigned to C-1′, and 175.2–179.9 for the carboxylic acid group. For compounds **3** and **4,** signals for the acetamide (CH_3_CONH-) were found ranging from δ_C_ 23.8 to 24.5 ppm for the methyl group and 167.9 to 168.4 ppm for the carbonyl group.

### 2.2. In Vitro PPARα/γ and GLUT-4 Expression

Initially, the viability of 3T3-L1 fibroblasts were studied at increasing concentrations of 1, 10, and 100 μM of compounds **1**–**4** using the MTT assay, and no cytotoxicity was observed. For the in vitro mRNA expression of PPARα, PPARγ, and GLUT-4, murine fibroblasts were differentiated to adipocytes to detect the action of molecules on the expression of genes. Cells were treated for 24 h with 10 μM of compounds **1**–**4**. Pioglitazone (PIO) and clofibrate (CLO) were used as positive controls [8,9].

**Figure 3:**
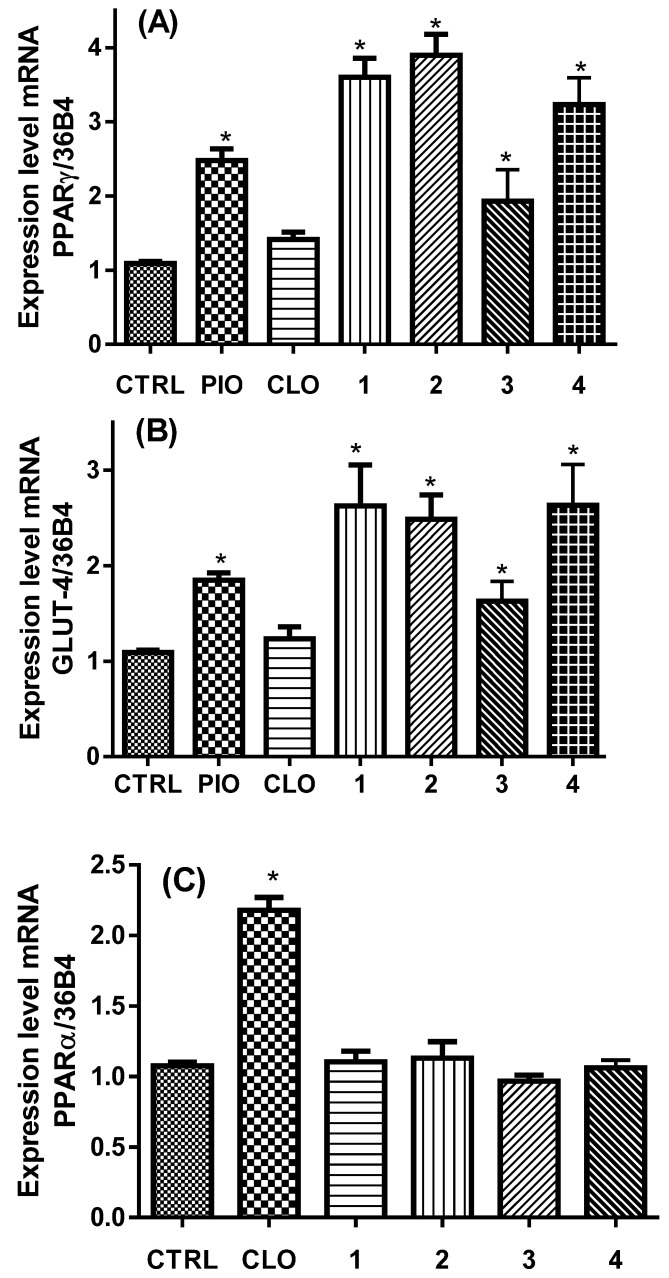
Changes on the expression of PPARγ (**A**), GLUT-4 (**B**), and PPARα (**C**) genes induced by pioglitazone, clofibrate, and isobutyric acid derivatives **1**–**4**. * Statistically significant difference between control group and test samples were estimated using ANOVA with post hoc Dunnett’s test (*n* = 5, mean ± SEM, *p* < 0.001 compared with CTRL group).

The variation in the mRNA expression levels was estimated by qPCR. Figure 3 shows that compounds **1**–**4** unexpectedly augmented with statistical significance the mRNA expression of PPARγ (around two- to four-fold) and its downstream gene GLUT-4 (three-fold), as pioglitazone (PIO) did. PPARγ modulation decreases glycemia in diabetic individuals through an enhancement in insulin sensitization, and the rise in GLUT-4 concentrations in striated muscle is critical for glucose homeostasis. These unexpected results found in this study suggest that nitrocompounds **1**, **2**, and acetamide **4** stimulates GLUT-4 mRNA concentration greater than pioglitazone (Figure 3B).

As expected, clofibrate (CLO) did not provoke a statistically significant rise in the mRNA expression level of PPARγ nor in GLUT-4 expression. Conversely, none of the compounds had activity over PPARα expression, whereas clofibrate did. This implies that azasubstituents (nitro and acetamide), instead of the chlorine atom in the phenyl ring, provoke a selectivity of compounds over PPARγ instead PPARα and are able to behave as selective PPAR modulators (SPPARM) [10].

### 2.3. In Vivo Antidiabetic Action

**Figure 4:**
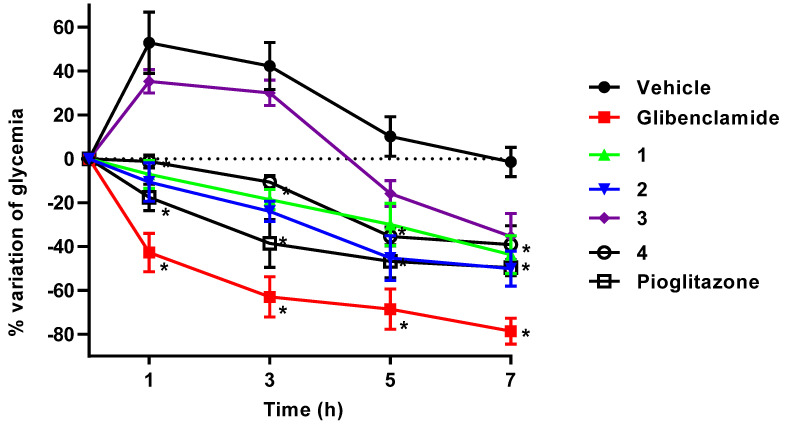
Effect of a intragastric single dose of 100 mg/kg of (thio)phenoxyisobutyric derivatives **1**–**4**, glibenclamide, and pioglitazone on blood glucose levels in STZ/NA-induced diabetic mice. * Statistically significant difference between control group and test samples were estimated using ANOVA and a multiple comparison Dunnett’s test (*n* = 6, mean ± SEM, *p* < 0.05).

To confirm the possible hypoglycemic and/or antihyperglycemic action of compounds **1**–**4**, we performed an in vivo acute experiment in streptozotocin (STZ)- and nicotinamide (NA)-induced diabetic mice, employing glibenclamide (Gli) as a hypoglycemic control in order to ensure that the damage to β-cells was partial and that the mice’s pancreases still produced insulin and responded to a secretagogue drug. Additionally, we used pioglitazone as a positive control of an antihyperglycemic drug mediated by the PPARγ mechanism. Figure 4 shows the results of this experiment. Compounds **1**, **2**, and **4** administered at 100 mg/kg via intragastric route considerably decreased glycemia sixty minutes after oral administration and maintained their glycemic-lowering effect throughout the experiment in comparison with the vehicle (Tween 80, 10%). During the assay, glycaemia did not fall further than normal levels, as glibenclamide did, indicating that compounds **1**–**4** had an antihyperglycemic effect in agreement with insulin sensitization triggered by PPARγ activation [11], since the behavior of pioglitazone during the experiment was the same. It is important to note that compound **3** did not show a glycemic reduction effect until 5 and 7 hours post administration, its effect attributable to a possible prodrug of **4**, which would need to be bioactivated by phase I hydrolysis within the murine organism.

### 2.4. Molecular Docking Calculations

**Figure 5:**
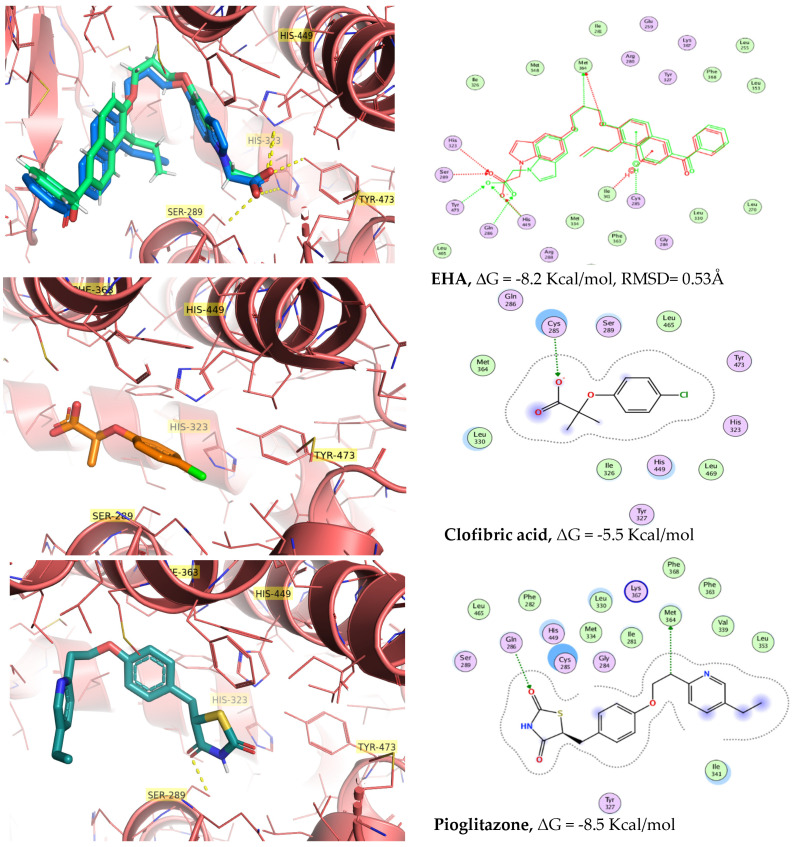
Three-dimensional pose of co-crystal structure reference of 2F4B (PPARγ)/EHA (carbon atoms colored blue, X-ray; and green, redocked pose). Docked pose of clofibric acid (carbon atoms colored orange), pioglitazone (carbon atoms colored cyan), and its 2D diagram of interaction. (In the case of EHA, the 2D overlay complexes are displayed in green for the co-crystal X-ray representation and red for the re-docked pose.)

**Figure 6:**
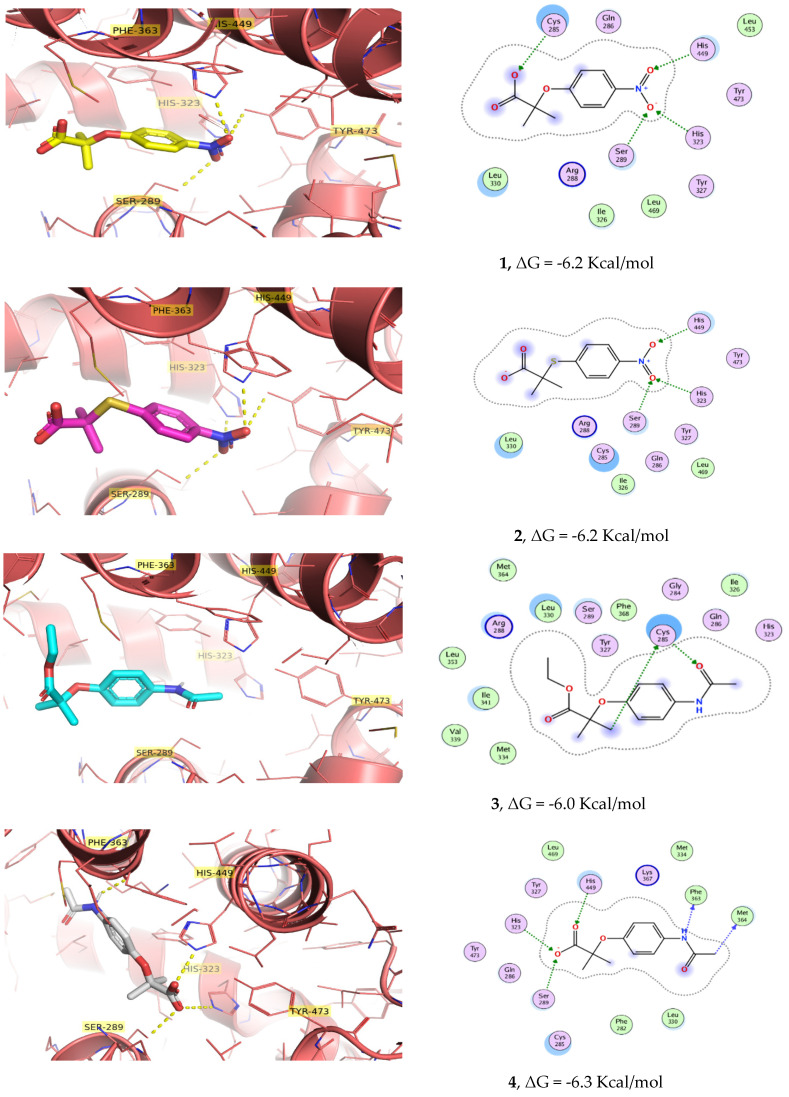
Docked poses of **1** (carbon atoms colored yellow), **2** (carbon atoms colored magenta), **3** (carbon atoms colored cyan), and **4** (carbon atoms colored gray), and 2D diagram of interactions in the ligand-binding site of PPARγ.

Once the in vitro activation of PPARγ and GLUT-4, as well as the in vivo antihyperglycemic effects, had been corroborated, compounds **1**–**4** were subjected to an in silico docking simulation. Docking calculations showed that molecules **1**–**4** enter into the ligand-binding pocket of PPARγ (PDB ID: 2F4B) and produce a net of hydrogen bonds with His-323 and several polar contacts with Cys-285, Tyr-327, Tyr-473, and His-449, key residues for the PPARγ activation (Figure 5 and Figure 6). In the same way, (5-{3-[(6-benzoyl-1-propyl-2-naphthyl)oxy]propoxy}-1*H*-indol-1-yl)acetic acid (**EHA**) was previously co-crystallized with PPARγ and shared the same contacts [12]. However, compound **3** had none of these types of relevant interactions, which correlates with the low activity shown both in vitro and in vivo studies. It is important to mention the participation of nitro group-containing compounds **1** and **2** in the network, generating polar contacts with His-449, His-323, and Ser-289. Clofibric acid was also docked in PPARγ for comparative purposes with the 4-azasubstituted-(thio)phenoxyisobutyric acids, showing less affinity than **1**–**4**. Validation of docking yielded an RMSD value of 0.53 Å^2^. PyMol and MOE [13] were employed for visualization.

Compound **3** (an ethyl ester) displayed a different conformation than its hydrolyzed product **4** (carboxylate ionized form). Although the benzene group was properly oriented to interact with the His-327 and Ser-289, the part of the ethyl ester was adapted to the side arm of the PPAR cavity (Figure 6). Molecular docking binding energies and calculated affinities (*K_i_*) on PPARγ agree with the behavior of compounds **1**–**4** and clofibrate in the in vitro and in vivo pharmacological screens (Table 1), suggesting that the antihyperglycemic effect displayed is mediated by insulin sensitization.

### 2.5. Molecular Dynamics Simulations

**Figure 7:**
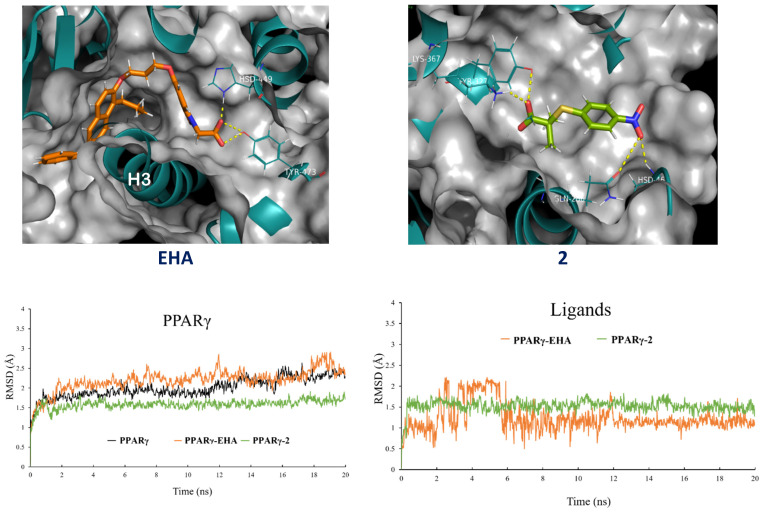
Hydrogen-bonding network of **EHA** (orange) and nitrocompound **2** (green) in the binding pocket of PPARγ (PDB code: 2F4B); RMSD of protein and ligands.

The molecular dynamics simulation was used to analyze and understand the dynamic motion and the degree of stability of the complexes. The RMSD of the protein backbone and ligands is illustrated in Figure 7, where the complexes of nitrocompound **2** had initial stability after 2 ns. Interestingly, the two first complexes did not reveal RMSD values over 1.5 Å, which corroborates the rigorous conformation of the most active complexes. These complexes maintained a stable RMSD profile till the rest simulation. In Figure S1 (see Supplementary Materials), it was also observed that protein coupled to compound **4** and clofibric acid had similar RMSD profiles from 8 to 20 ns. Unlike the backbone of EHA and pioglitazone complexes, the RMSD profile indicates the degree of protein folding and unfolding importantly until reaching a semi-equilibrium at the end of the simulation. In contrast, in the values of the RMSD profile for the ligands, greater stability and less variation can be observed in terms of their structural conformation; however, the RMSD values for compounds **1**, **2**, and **4** oscillate between 1.5 after one nanosecond of simulation, which indicates that they easily find their conformation and stability in the active site, anchoring themselves to nearby amino acid residues previously predicted during the molecular docking study (see Supplementary Materials). This helps us to corroborate and to validate the molecular docking study. Finally, from Figure 7 we can observe that there is a conservation of anchoring with amino acids Lys-367, Tyr-327, and His-449 that retain most of the compounds. However, nitrocompound **2** showed an extra interaction with Gln-286. After 2 ns, compound **2** was anchored with its nitro group to Gln-286, His-466, and the carboxylate head with Lys-367 and Tyr-327 during 20 ns of simulation. Distinct to the rest of the compounds, nitroderivative **2** interacts with Gln-286, which undergoes a side chain reorientation in the complex, and it is evident that Gln-286 plays a key role in stabilizing helix 3 and 12 of PPARγ and has an important impact on receptor activity [14]. Taking into account that the partial modulators preferentially stabilized the β-sheet and H3 to a larger degree [15], nitrocompound **2** could belong to the partial modulators and it would show fewer adverse effects. Furthermore, the activity shown by this compound in the in vivo and in vitro models correlates with this greater stability found in the molecular dynamics studies and its extra anchoring with Gln-286. In addition, we decided to use another method to obtain the free binding energy of the molecular dynamics complex (PPARγ/*MD*). The PRODIGY-LIG web server [16] uses a refinement protocol in order to collect intermolecular energy and it is, therefore, suitable for every type of complex. To use the PRODIGY tools, we just needed to provide the 3D structure of our complex or complexes in PDB format and the ID. The PRODIGY-LIG web server predicts binding affinities with an accuracy of 1.89 kcal/mol (RMSE). The results are summarized in Table 1. To obtain an outstanding compound, we analyzed and carried out the calculation to determine predictive binding energies through the PRODIGY-LIG web server [17]. The values obtained after submitting the complexes were approximate to free binding energies found in molecular docking, with slightly more negative values indicating a stronger binding along with molecular dynamics. The development of more balanced drugs interacting with PPARs, devoid of the side-effects shown by the currently marketed PPARγ full agonists, is considered the major challenge in drug design. For this reason, an alternative approach for the treatment of metabolic disorders is represented by the development of partial PPARγ agonists or selective PPARγ modulators (SPPARM), such as the compounds presented herein.

### 2.6. In Silico Toxicology

It is well known that drugs containing nitro groups can induce severe idiosyncratic toxicity and this is definitely the reason in many cases for their being avoided in drug design, considered as structural alerts [18]. However, the nitro group is also considered as both a pharmacophore and a selective toxicophore associated with organ-selective toxicity [19]. With the purpose of anticipating latent toxicity problems of compounds **1**–**4**, a computational simulation of security profiles was performed. The toxicity parameters of **1–4,** clofibrate, and pioglitazone were calculated employing ACD/ToxSuite, v. 2.95 (Table 2).

The in silico calculation of inhibition for the three main isoforms of CYP450 for compounds **1**–**4** were comparable to clofibrate at relevant clinical concentrations <10 μM, showing extremely low probabilities of drug–drug interactions and undesirable adverse effects. Compounds **1**–**4** showed very low prediction of hERG channel blockage at clinically relevant concentrations (*K_i_* < 10 μM), being considered as non-cardiotoxic molecules. In the calculation of acute toxicity, compounds **1**–**4** revealed similar LD_50_ values to clofibrate and pioglitazone by two different administration routes in two murine models. It is important to note that experimentally the viability of 3T3-L1 fibroblasts did not show any cytotoxicity provoked by compounds **1**–**4** assayed at 100 μM concentration.

The term ‘cliffs of toxicity’ was coined in 2015 by our group [20] and taken up again in 2019 by other researchers [21] to define various nitrocompounds that have been reported as mutagenic and carcinogenic drugs (e.g., metronidazole, nimesulide, flutamide, nitazoxanide, among others) according to red flags included in most of the in silico tools that predict potentially toxic substructures and that nevertheless have a very low experimental toxicity [20]. In current work, compounds **1** and **2** could be considered toxicity cliffs due to the demonstrated lack of toxicity, in spite of the presence of a red flag in its structure.

On the other hand, in order to evaluate the pharmacokinetic profile of compounds **1**–**4**, we used the platform ADMETLab 2.0 [22] to calculate the ADME profile (Table 3), starting with absorption parameters: GI absorption, blood–brain barrier permeability, and bioavailability.

Compounds **1**–**4** present high values of absorption and only compound **3** and clofibrate are able to cross the BBB. The distribution parameters calculated were plasma protein binding and volume of distribution. Optimal plasma protein binding is less than 95% and all compounds met this parameter. However, compounds with high protein binding may have a low therapeutic index, such as clofibrate. Metabolic stability was also predicted, and compounds **1**–**4** are substrates of CYP450, which implies that this enzyme is the main metabolizing protein for most of the drugs that enter the body. To finish the ADME calculations, the excretion parameters were predicted, the compounds showing satisfactory values of clearance and long half-lives (>3 h) compared with clofibrate.

## 3. Materials and Methods

### 3.1. Chemistry

All reagents were purchased from Merck^®^ (Darmstadt, Germany) and were used without any extra purification. Using a Variant Oxford Instrument (Palo Alto, CA, USA, 600 MHz and 150 MHz, respectively), ^1^H and ^13^C nuclear magnetic resonance spectra were obtained. Molecular masses were obtained with a JMS-700 spectrometer (JEOL, Tokyo, Japan) with an impact electronic method. Melting points were obtained using an EZ-Melt MPA120 automated apparatus from Stanford Research Systems (Sunnyvale, CA, USA).

#### 3.1.1. Procedure for the Synthesis of Compounds **1**–**4**

##### 2-(4-Nitrophenoxy)isobutyric Acid (**1**)

To a mixture of 4-nitrophenol (1.0 g, 4.44 mmol), potassium carbonate (1.22 g, 8.88 mmol) in acetonitrile, ethyl 2-bromo-2-methylpropionate (1.29 g, 1.04 mL, 6.66 mmol) was added dropwise. The mixture was stirred and heated under reflux for 8 h, then poured onto cold water. The resulting oil was treated with a mixture of THF/MeOH/H_2_O (3:2:1 *v*/*v*/*v*), LiOH (5 equiv.) was added, and the mixture was stirred at room temperature for 3 h, then 10% HCl solution was added and most of the organic solvents removed. The solid residue was extracted with dicloromethane (3 × 10 mL) and the combined organic layers were dried with Na_2_SO_4_, filtered, and concentrated to give a yellow solid which was recrystallized from chloroform, m.p. 122.8–124.3 °C [23,24]; yield 62.8%. ^1^H NMR (200 MHz, CDCl_3_) δ:1.71 (s, 6H, (CH_3_)_2_), 6.91 (d, 2H, H-2′, H-6′, *J* = 9.2 Hz), 8.16 (d, 2H, H-3′, H-5′, *J* = 9.6), 10.60 (s, 1H, CO_2_H) ppm. ^13^C NMR (50 MHz, CDCl3) δ: 25.5 ((CH_3_)_2_), 79.8 (C-2), 118.2 (C-2′, C-6′), 125.7 (C-3′, C-5′), 142.3 (C-4′), 160.6 (C-1′), 179.1 (COOH) ppm. MS/EI: *m*/*z* (% rel. int.). 225 (M^+^, 1%), 180 (M-45, 100%). Anal. calcd. for C_10_H_11_NO_5_: C, 53.33; H, 4.92; N, 6.22. Found: C, 53.41; H, 4.83; N, 6.28.

##### 2-(4-Nitrophenylsulfanyl)isobutyric Acid (**2**)

To a mixture of 4-nitrothiophenol (1.0 g, 6.40 mmol), potassium carbonate (1.94 g, 14.1 mmol) in acetonitrile, ethyl 2-bromoisobutyrate (1.37 g, 7.04 mmol) was added dropwise. The mixture was stirred and heated under reflux for 6 h. After that, the mixture was poured onto cold water. The resulting oil was treated with a mixture of THF/MeOH/H_2_O (3:2:1, *v*/*v*/*v*), and LiOH was added (5 equiv.). The mixture was stirred at room temperature for 3 h. Then, HCl solution (10% *v*/*v*) was added, and most of the organic solvents removed. The solid residue was extracted with dichloromethane (3 × 10 mL) and the combined organic layers were dried with Na_2_SO_4_, filtered, and concentrated to give a yellow solid which was recrystallized from chloroform, m.p. 121.9–123.7 °C [25,26]; yield 58.9%. ^1^H NMR (400 MHz, CDCl_3_) δ: 1.56 (s, 6H, (CH_3_)_2_), 7.65 (dd, 2H, H-2′, H-6′, *J* = 2.6, *J* = 9.6 Hz), 8.17 (dd, 2H, H-3′, H-5′, *J* = 2.6, *J* = 9.6 Hz), 10.60 (s, 1H, CO_2_H) ppm. ^13^C NMR (100 MHz, CDCl_3_) δ: 25.9 ((CH_3_)_2_), 51.5 (C-2), 123.8 (C-2′, C-6′), 136.1 (C-3′, C-5′), 140.6 (C-1′), 145.4 (C-4′), 179.9 (COOH) ppm. MS/EI: *m*/*z* (% rel. int.). 241 (M^+^, 1%), 196 (M-45, 100%). Anal. calcd. for C_10_H_11_NO_4_S: C, 49.78; H, 4.60; N, 5.81; S, 13.29. Found: C, 49.69; H, 4.60; N, 5.87; S, 13.36.

##### Ethyl 2-[4-(acetylamino)phenoxy]isobutyrate (**3**)

In the minimum amount of dimethylsulfoxide, 4-Acetylaminophenol (1 g, 6.6 mmol) and potassium carbonate (2 g, 14 mmol) were dissolved and heated at 40 °C. After 20 min, the ethyl 2-bromo-2-methylpropionate (1.45 mL, 9.9 mmol) was added dropwise and the reaction mixture was heated to reflux (80 °C) and monitored by TLC. After the reaction completion (15 h), the mixture was filtered and the solid residue was recrystallized from acetone. White crystals, m.p. 90.1–92.3 °C [27]; yield 44.0%. ^1^H NMR (200 MHz; DMSO-d_6_; Me_4_Si) δ: 1.17 (3H, t, CH_3_), 1.47 (6H, s, (CH_3_)_2_), 2.00 (3H, s, CH_3_CO), 4.15 (2H, q, CH_2_), 6.75 (2H, d, H-2′, H-6′, *J = 8.7* Hz), 7.45 (2H, d, H-3′, H-5′, *J* = 8.7 Hz), 9.83 (1H, bs, N–H) ppm. ^13^C NMR (50 MHz, DMSO-d_6_) δ: 14.6 (CH_3_), 24.5 (CH_3_CO), 25.7 ((CH_3_)_2_), 61.6 (C-2), 79.5 (CH_2_-O), 120.3 (C-2′, C-6′), 120.7 (C-3′, C-5′), 134.7 (C-4′), 150.9 (C-1′), 168.4 (NHC=O), 173.7 (O-C=O) ppm. EI-MS: *m*/*z* (rel. int.) 265 (M^+^, 25%), 192 (25%), 151 (50%), 109 (100%). Anal. calcd. for C_14_H_19_NO_4_: C, 63.38; H, 7.22; N, 5.28. Found: C, 63.38; H, 7.07; N, 5.53.

##### 2-(4-Acetamidophenoxy)isobutyric Acid (**4**)

Compound **3** (0.5 g, 1.88 mmol) was treated with a mixture of tetrahydrofuran/H_2_O (5:1, *v*/*v*) and LiOH was added (3 equiv.). The mixture was stirred at room temperature for 3 h. Then, HCl solution (10% *v*/*v*) was added and most of the organic solvents removed. The solid residue was extracted with CH_2_Cl_2_ (3 *×* 10 mL), dried with Na_2_SO_4_, filtered, and concentrated to give a white solid which was recrystallized from methanol, m.p. 164.4–166.3 °C [28]; yield 83.13%. ^1^H NMR (200 MHz; DMSO-*d*_6_; Me_4_Si) δ 1.46 (6H, s, (CH_3_)_2_), 2.10 (3H, s, CH_3_CO), 6.78 (2H, d, H-2′, H-6′, *J* = 8.7 Hz), 7.44 (2H, d, H-3′, H-5′, *J* = 8.7 Hz), 9.83 (2H, bs, N–H, COOH) ppm. ^13^C NMR (50 MHz, DMSO-*d*_6_) *δ*: 23.8 (CH_3_CO), 25.1 ((CH_3_)_2_), 78.7 (C-2), 119.5 (C-2′, C-6′), 120.2 (C-3′, C-5′), 133.9 (C-4′), 161.8 (C-1′), 167.9 (NHC=O), 175.2 (COOH) ppm. EI-MS: *m/z* (rel. int.) 237 (M^+^, 5%), 192 (M-45, 100%). Anal. calcd. for C_12_H_15_NO_4_: C, 60.75; H, 6.37; N, 5.90. Found: C, 60.8; H, 6.39; N, 5.85.

### 3.2. Biological Assays

#### GLUT-4 and PPAR Quantification

Confluent cultures of 3T3-L1 fibroblasts were differentiated to the adipocytes, employing 0.25 μM of dexamethasone acetate, 0.5 mM of 3-isobutyl-1-methylxanthine, and 0.8 μM bovine insulin. After 10 days, the cells gained the matured adipocyte phenotype and were preserved for 24 h to observe the effects of cumulative concentrations of **1**–**4** on GLUT-4 and PPARγ mRNA expression levels. Total mRNA was isolated from adipocytes, and 2 μg was transcribed by RT-PCR. The cDNA was amplified using SYBR Green Master Mix containing 0.5 mM of customized primers for GLUT-4 (GenBank accession: NM009204.2), PPARγ (GenBank accession: NM011146.1), and PPARα (GenBank accession: NM011144). PCR was used for individually sampling and calculating the threshold cycles (Ct) and the ΔCt values. The quantity of mRNA for each gene was normalized according to the amount of mRNA encoding ribosomal protein 36B4 (Gene Bank NM007475.2). The ΔCt values were calculated in every sample for each gene of interest. Oscillations in the relative expression levels of independently specific genes (ΔΔCt) were measured and graphed [8,9].

### 3.3. In Vivo Antidiabetic Assay

#### 3.3.1. Animals

ICR male mice weighing 25 ± 5 g were housed in animal cages with periods of 12 h light and 12 h dark. Mice were maintained at 25 °C environment, with water and food access ad libitum. All mouse experiments were conducted according to protocols that were approved by the Mexican government NOM-065-ZOO-1999 and NOM-033-ZOO-2014 and ratified by the Institutional Ethics Committee of the Universidad Autónoma Metropolitana (dictum 1857), based on US National Institutes of Health Publication #85-23, revised 1985.

#### 3.3.2. Acute Antidiabetic Assay

Induction of diabetes was executed agreeing to earlier works [5,8]. Mice were fasted for 8 h. Nicotinamide was dissolved in saline water and administered i.p. at a dose of 20 mg/kg. Fifteen minutes later, streptozotocin was administered i.p. in a citrate buffer 0.05 M, pH = 4.5, at a dose of 100 mg/kg. Mice that developed blood glucose levels over 180 mg/dL were selected for the in vivo assay. The animals were fasted for 8 h and the groups’ (*n* = 6) doses were administered orally, 100 mg/kg of the different compounds, including one vehicle group (10% Tween 80) and two positive control groups (glibenclamide, 20 mg/kg; pioglitazone, 30 mg/kg). Blood glucose concentrations were measured at 0, 1, 3, 5, and 7 h post administration, employing a blood sugar monitoring device (Accu-Check Performa).

### 3.4. In Silico Docking Calculations

In silico calculations were performed with Autodock Vina [29]. The crystal structure of PPARγ (pdb id: 2F4B), complexed with EHA at 2.07 Å resolution, was obtained from the Protein Data Bank (http://www.rcsb.org/pdb accessed on 5 January 2021). All water molecules were deleted, non-polar hydrogen atoms were merged, and the hydrogens atoms and Gasteiger charges were added in MGLTools 1.5.4 [30]. The 3D structures were built, minimized, and ionized in MOE, using the MMFf94 forcefield [13]. The grid was centered at the crystallographic coordinates of EHA (center_x = 8.693; center_y = −6.961; and center_z = 39.672) and the grid dimensions were 40 × 40 × 40 points with 0.375 Å of spacing. One hundred independent Genetic Algorithm runs from AutoDock were processed using the built-in clustering analysis with a 2.0 Å cutoff. In concordance with a validation procedure to reproduce by docking, the same pose of co-crystallized ligand in the crystal structure (RMSD = 0.53 Å) was used, with a score of −8.2 Kcal/mol. After the molecular docking, we analyzed the best calculated binding poses, and the graphical representations were performed by Surface Maps and Ligand Interaction from MOE [13] and PyMol software.

#### Docking Validation

The docking protocols were validated by EHA (co-crystal ligand) into the PPARγ ligand-binding site of 2F4B. The root mean square deviation (RMSD) between the co-crystal ligand and the redocked molecule was 0.53 Å, specifying that the parameters for docking calculations are replicating conformation and orientation in the X-ray crystal of PPARγ.

### 3.5. Molecular Dynamics Simulations

The obtained complexes of compound **1**–**4**, clofibric acid, pioglitazone, and EHA with PPARγ protein (PDB code: 2F4B) were used to perform the molecular dynamics simulations. The objective of this work was to evaluate the stability and behavior of ligand–receptor complexes previously tested in in vitro and in vivo studies, with the earlier published protocol [31].

### 3.6. Statistical Analysis

To analyze the differences in the percent variation of glycemia and the in vitro PPARγ and GLUT-4 quantification, we employed ANOVA, complemented with a Dunnett’s multiple test. All values were expressed as the mean ± S.E.M. *p* < 0.05, using GraphPad Prism 5.0 for the analysis.

## 4. Conclusions

In summary, four 4-azasubstituted (thio)phenoxyisobutyric acids have been synthesized and they significantly increased mRNA expression of PPARγ and GLUT-4, to a greater extent than clofibrate and pioglitazone. All of them showed selective activation of PPARγ over PPARα. Their main difference with clofibrate is the absence of the chlorine atom in position 4 of the benzene ring and the incorporation of azasubstituents, such as nitro (compounds **1, 2**) and acetamide groups (compounds **3, 4**). Compounds **1, 2**, and **4** also showed oral antihyperglycemic effects in the diabetic rat model, consistent with insulin sensitization and with their behaving as selective PPARγ modulators (SPPARM), such as pioglitazone. Finally, based on the findings of molecular dynamics simulations, nitrocompound **2** proved outstanding for its stability in the ligand–receptor complex and could belong to the partial modulators due to its extra anchoring formed with its nitro group and Gln-286.

## Figures and Tables

**Table 1:** Molecular docking binding energies, calculated affinities, molecular dynamics binding energies, and quantitative pharmacological actions for compounds **1**–**4**, clofibric acid, and pioglitazone.

Compound	PPARγ ΔG (kcal/mol)	*K_i_* (μM)	PPARγ*/MD* ΔG (kcal/mol)	PPARγ Expression Level (Fold)	GLUT-4 Expression Level (Fold)	Maximal Percentage of Glycemic-Lowering Effect (%)
**1**	−6.2	5.39	−7.3	3.60	2.62	−43.5
**2**	−6.2	3.90	−7.8	3.89	2.48	−50.1
**3**	−6.0	7.54	−7.2	1.92	1.62	−35.3
**4**	−6.3	4.28	−6.9	3.23	2.63	−39.1
**Clofibric acid**	−5.5	13.13	−6.8	1.41	1.02	No reduction observed [5]
**Pioglitazone**	−8.5	0.50	−9.7	2.51	1.84	−49.6

**Table 2:** Predicted toxicity profiles calculated for compounds **1–4,** clofibrate, and pioglitazone.

Compound	LD_50_ (mg/kg)				Probability of Inhibition/Blockage (IC_50_ or *K_i_* < 10 μM)			
	Mouse		Rat		CYP450 Isoform			hERG
	i.p.	p.o.	i.p.	p.o.	3A4	2D6	1A2	
**1**	680	1500	820	1590	0.00	0.00	0.00	0.01
**2**	670	790	520	1280	0.00	0.01	0.00	0.01
**3**	810	1900	770	3000	0.02	0.01	0.19	0.05
**4**	790	1400	970	2500	0.00	0.00	0.00	0.01
**Clofibrate**	750	1300	1200	1800	0.03	0.04	0.31	0.12
**Pioglitazone**	400	1400	400	1100	0.22	0.03	0.02	0.10

**Table 3:** Pharmacokinetic predictive values calculated with ADMETLab 2.0 (https://admetmesh.scbdd.com/ accessed on 5 March 2021) for compounds **1**–**4** and clofibrate.

	Model	Compounds				
		1	2	3	4	Clofibrate
A	Gastrointestinal Absorption	(+) High	(+) High	(+) High	(+) High	(+) High
	Blood–Brain Barrier permeant	(−) No	(−) No	(+) Yes	(−) No	(+) Yes
	Bioavalability (F)	<20%	>30%	>30%	>30%	>30%
D	Plasma Protein Binding	90.62%	91.22%	60.41%	42.14%	97.21%
	Volume distribution	0.23 L/kg	0.32 L/kg	0.95 L/kg	0.41 L/kg	1.403 L/kg
M	CYP3A4 substrate	(+) Yes	(+) Yes	(++) Yes	(+) Yes	(++) Yes
	CYP2D6 substrate	(−) No	(−) No	(+) Yes	(−) No	(+) Yes
E	Clearance (Cl)	0.948 mL/min/kg	0.412 mL/min/kg	5.860 mL/min/kg	1.093 mL/min/kg	5.202 mL/min/kg
	Half Life(T_1/2_)	>3 h	>3 h	>3 h	>3 h	>3 h

## Data Availability

Data is contained within the article and Supplementary Files.

## Supplementary Materials

The following are available online at https://www.mdpi.com/article/10.3390/ph15010102/s1, Figure S1: Hydrogen-bonding network of EHA (orange), Pioglitazone (pink), Clofibric acid (yellow) and compounds 1-4 (red, green, deep blue and cyan, respectively) in the binding pocket of PPARγ (PDB code: 2F4B); RMSD of Protein and Ligands.

## Sample Availability

Samples of the compounds are available from the authors.
