# Supplementary material for: Synthesis, In Vitro, In Vivo and In Silico Antidiabetic Bioassays of 4-Nitro(thio)phenoxyisobutyric Acids Acting as Unexpected PPARγ Modulators: An In Combo Study
Source: Pharmaceuticals (Basel). 2022 Jan 15;15(1):102. doi: 10.3390/ph15010102 (PMC8779174; doi:10.3390/ph15010102)
Supplement: Supplementary file 1 [file pharmaceuticals-15-00102-s001.zip › pharmaceuticals-1550172-supplementary.pdf]

Supplemental info

Synthesis, *in vitro*, *in vivo* and *in silico* antidiabetic bioassays of 4-nitro(thio)phenoxyisobutyric acids acting as unexpected PPARγ modulators: an *in combo* study

Blanca Colin-Lozano,[1,2] Héctor Torres-Gomez,[1,3] Sergio Hidalgo-Figueroa,[1,4] Fabiola Chavez-Silva,[1] Samuel Estrada-Soto,[1] Julio Cesar Almanza-Pérez,[5] and Gabriel Navarrete-Vazquez [1,*]

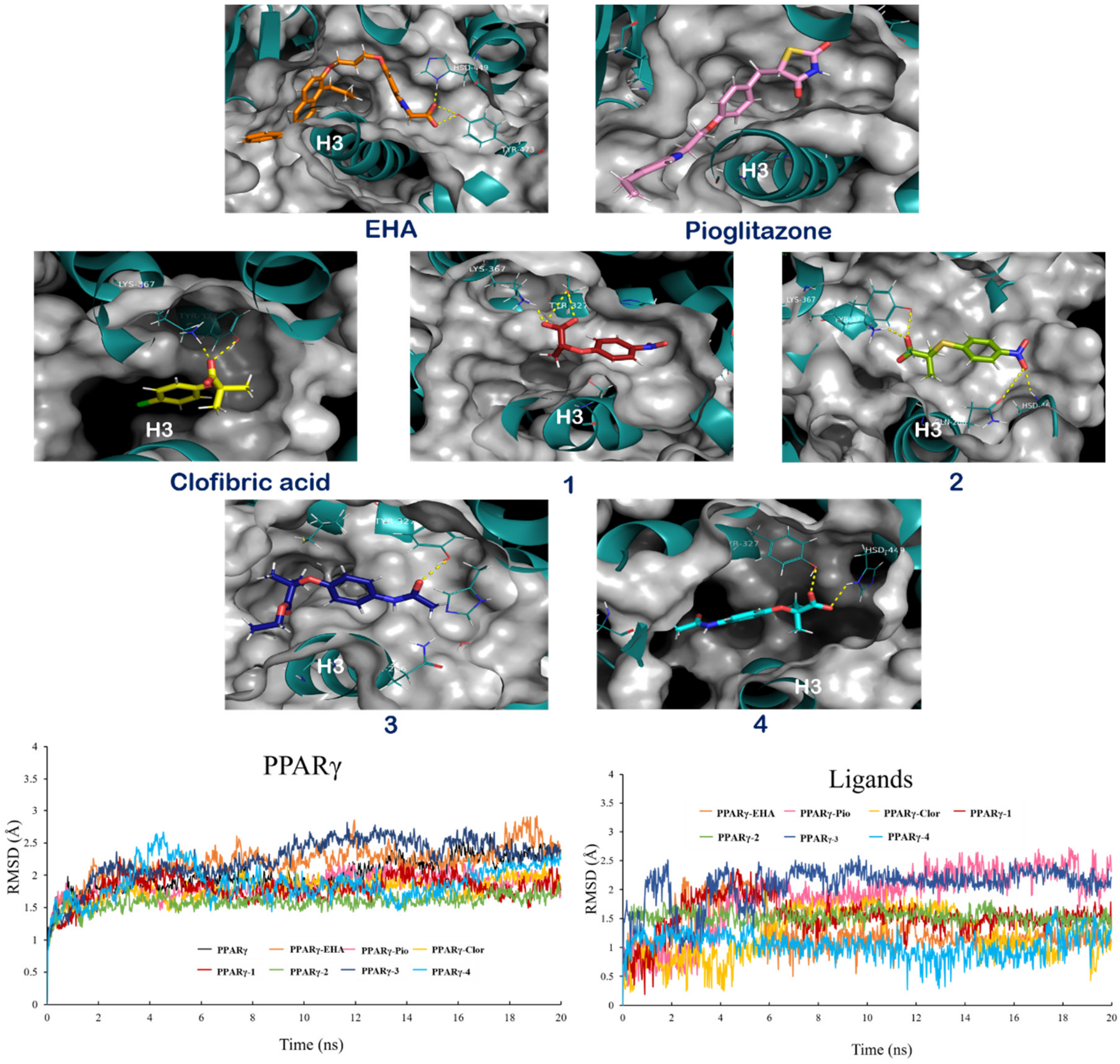

Figure S1. Hydrogen-bonding network of **EHA** (orange), **Pioglitazone** (pink), **Clofibric acid** (yellow) and compounds **1-4** (red, green, deep blue and cyan, respectively) in the binding pocket of PPARγ (PDB code: 2F4B); RMSD of Protein and Ligands.
